# Efficiency of True-Green Light Emitting Diodes: Non-Uniformity and Temperature Effects

**DOI:** 10.3390/ma10111323

**Published:** 2017-11-18

**Authors:** Ilya E. Titkov, Sergey Yu. Karpov, Amit Yadav, Denis Mamedov, Vera L. Zerova, Edik Rafailov

**Affiliations:** 1 Ostendo Technologies Inc., 6185 Paseo del Norte, Carlsbad, CA 92011, USA; Denis.mamedov@ostendo.com; 2 Optoelectronics and Biomedical Photonics Group, Aston Institute of Photonic Technologies, Aston University, Birmingham B4 7ET, UK; a.yadav1@aston.ac.uk (A.Y.); vzerova@gmail.com (V.L.Z.); e.rafailov@aston.ac.uk (E.R.); 3 STR Group—Soft-Impact, Ltd., P.O. Box 83, 27 Engels Ave., 194156 Saint-Petersburg, Russia; sergey.karpov@str-soft.com; 4 Nanoscale Physics Research Laboratory, School of Physics and Astronomy, University of Birmingham, Birmingham B15 2TT, UK

**Keywords:** InGaN green LEDs, active region non-uniformity, temperature-dependent electroluminescence, internal quantum efficiency, light extraction efficiency, extended defects, modeling

## Abstract

External quantum efficiency of industrial-grade green InGaN light-emitting diodes (LEDs) has been measured in a wide range of operating currents at various temperatures from 13 K to 300 K. Unlike blue LEDs, the efficiency as a function of current is found to have a multi-peak character, which could not be fitted by a simple ABC-model. This observation correlated with splitting of LED emission spectra into two peaks at certain currents. The characterization data are interpreted in terms of non-uniformity of the LED active region, which is tentatively attributed to extended defects like V-pits. We suggest a new approach to evaluation of temperature-dependent light extraction and internal quantum efficiencies taking into account the active region non-uniformity. As a result, the temperature dependence of light extraction and internal quantum efficiencies have been evaluated in the temperature range mentioned above and compared with those of blue LEDs.

## 1. Introduction

Semiconductor LEDs invented almost 100 years ago [1] have now become key components in numerous applications: solid-state lighting, traffic lights, brake lights, various indicators, and signs [2,3]. For many cases and, especially, for phosphor-free solid state lighting, high-efficiency green LEDs are of primary importance. Nowadays, a new level of external quantum efficiency (EQE), is achieved from commercial green LEDs: ~34% for directly emitting devices and ~54% for those using internal down-conversion of emitted light, see for example [4] that gives an overview of the external efficiency of different LED material systems of Osram OS. This progress is a result of both bandgap and strain engineering of the active multiple-quantum-well (MQW) regions, and optimized carrier transport across the LED structures and using advanced chip designs. 

Nevertheless, the EQE of green LEDs is still remarkably lower than that of blue LEDs, which is now over 70% at the emission wavelengths of 440–450 nm [4]. This fact is a manifestation of the so-called “green gap” problem, i.e., substantial efficiency reduction of InGaN-based LEDs from blue towards the green/orange spectral range. Among the mechanisms that may be responsible for the “green gap”, the ones that are frequently referred are: degradation of InGaN crystal quality at high indium content; polarization field built in the InGaN quantum wells (QWs), and carrier localization due to composition fluctuations in InGaN (see more detailed discussion given in Section 4.2). To better understand the role each factor plays in this problem, examination of temperature-dependent efficiency is quite constructive. 

This paper is a natural continuation of our previous study [5] in which temperature dependence of EQE, internal quantum efficiency (IQE), and light extraction efficiency (LEE) of an industrial-grade blue LED emitting at 440–450 nm has been determined in a wide range of temperatures from 13 K to 440 K. The study [5] was largely based on approximating the dome-like dependence of EQE on current/output optical power within a simple ABC recombination model. The subject of the present study is a green LED emitting in the range of 530–550 nm (so-called “true-green”). Recently, this method with minor modification has been extended to single-quantum well (SQW) LEDs operating at longer emission wavelengths [6]. However, EQEs of the green-emitting multiple quantum wells reported in [7,8] for a wide temperature range of 4–300 K did not exhibit a dome-like dependence on current, demonstrating more complex behavior that could not be fitted by a simple ABC-model. So, a more elaborate approach is required for experimental estimation of IQE and LEE from those or similar characterization data. Development of such an approach to evaluation of temperature-dependent efficiency of true-green LEDs is one of the goals of our study. 

Generally, blue LEDs exhibit a nearly evenly symmetrical dome-like EQE dependence on current/output power with respect to its value corresponding to the EQE maximum. In contrast, the EQE dependence of green LEDs frequently becomes asymmetric with a more extended low-current wing [9]. To explain that behavior, various mechanisms have been invoked: electron leakage into p-side of the LED structure [10]; delocalization of carriers captured by InGaN composition fluctuations [11]; imbalance between the electron and hole injection into InGaN QWs caused by non-equilibrium QW population [12], and suppression of non-radiative recombination at threading dislocations via carrier localization by composition fluctuations in InGaN alloys [13]. In addition, the contribution of device self-heating at high current was not reliably excluded in many experimental studies. Therefore, understanding the nature of the particular efficiency behavior of green LEDs is another goal of this study. 

Our data obtained with commercial true-green LEDs demonstrate non-ordinary variation of the emission wavelength with current and considerable deviation of EQE from a simple ABC-model. The low temperature measurements enable interpretation of the data in terms of the active region non-uniformity. The ABC-model modified for non-uniform QWs allow us to estimate the temperature-dependent LEE. 

## 2. Experimental

### 2.1. Samples and Characterization Techniques 

The LED samples and characterization techniques utilized herein were quite similar to those used in our previous research [5]. The LED structures were grown by metal-organic chemical vapor deposition on C-plane sapphire and consisted (from bottom to top) of an undoped GaN layer followed by a Si-doped n-GaN contact layer, undoped InGaN/GaN MQW active region, a p-AlGaN electron blocking layer, and Mg-doped p-GaN contact layer. The main difference from the previously studied blue LEDs was a higher, more than 20%, indium content in InGaN QWs, providing green light emission. The structures were processed as 1 × 1 mm^2^ UX:3 thin-film chips where high-reflective metallic electrodes were formed to the p-contact layer. After removing the sapphire substrate, the back (emitting) surface of the n-contact layer was textured to increase LEE, and the current access to the n-contact layer was provided by the metallic column electrodes passing through the 24 blind vias made in the structure and uniformly distributed over the emitting surface of the die [14]. The total area covered by vias did not exceed 0.5% of the emitting surface area. The chips were mounted onto the Dragon packages without any molding, which is suitable for temperature-dependent electroluminescence measurements. 

A spectrometer CDS-600 made by Labsphere Inc., North Sutton NH, UK; source-meter Keithley 2400 made by Tektronix Inc., Beaverton OR, USA and CCS-450 helium closed-cycle cryostat made by Janis, Woburn, MA, USA were used for the temperature- and current-dependent electroluminescence (T-I DEL) technique. In order to determine EQE, we measured electroluminescence (EL) over a wide range of operating current, from 20 nA to 800 mA. To cover the corresponding range of the output optical power, from 1 nW to 600 mW, we varied spectrometer exposure time from 1 ms to 5 s and used neutral ND1-4 filters. For calibration of EQE values, EL was measured both in an integrating sphere and in the cryostat at room temperature (RT). Then optical alignment was not changed at lower temperatures. The current–voltage (I–V) characteristics were measured with semiconductor characterization system Keithley 4200 made by Tektronix Inc., Beaverton, OR, USA that provided extended current range. 

### 2.2. Current-Voltage Characteristics 

**Figure 1:**
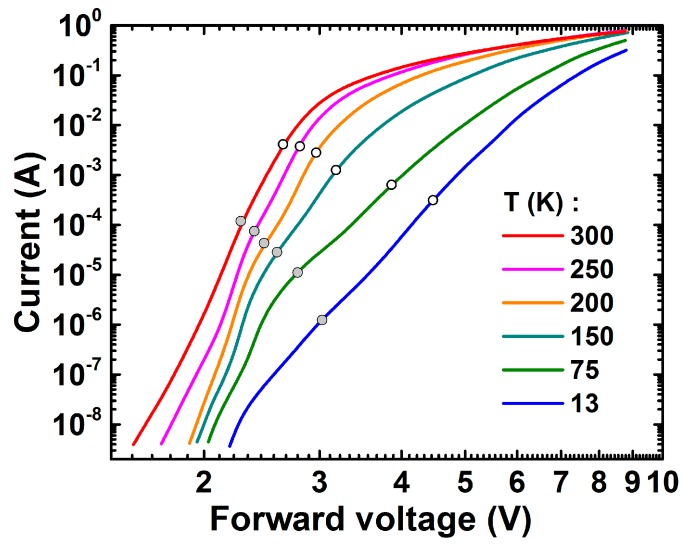
Current–voltage characteristics of the green LED measured at various temperatures. Closed and open circles indicate points at which shorter-wavelength and longer-wavelength emission approaches their maximum efficiency (see Section 2.4 for more details).

Current–voltage characteristics (I–V curves) of the green LED measured at various temperatures (13–300 K) are shown in Figure 1. As expected, every high temperature curve can be seen in two parts: the first one below ~3 V has a higher slope and can be associated with the carrier injection into the active region; the second one, above ~3 V, is controlled by the series resistance. No defect-mediated shoulders can be distinguished in the high-temperature I–V curves in contrast to those observed for blue LED [5]. However, one can see some traces of such shoulders in the low-temperature curves, looking like waving at the lower voltages (see also discussion on this issue in Section 4.3). 

Fitting of the I–V curves with Shockley’s diode equation accounting for the LED series resistance has shown that in the temperature range of 200–300 K the curves can be characterized by the ideality factor of ~3–5 and series resistance of ~7.3–7.6 Ω. For comparison, the ideality factors typical for blue LEDs [5] were about 1.7 with series resistance ~6.3–6.5 Ω. The relatively high ideality factor may be the evidence for impeded carrier transport over the barriers between the QWs [15], which correlates with much deeper InGaN QWs in green LEDs compared to blue ones, or high asymmetry in the donor and acceptor concentrations in the LED p-n junction [16]. 

At temperatures lower than 200 K, the character of the I–V curves changes dramatically. We believe that to originate from hole freezing out in the p-layers of LED structure because of a high activation energy of Mg acceptors. The reduction in the hole density in the p-GaN layer leads to less efficient thermionic emission or direct tunneling of holes across the metal–GaN interface. Moreover, an additional electric field in the p-GaN layer is required to maintain the current flow [17]. Identification of the carrier transport mechanism dominating at low temperatures requires, however, more detailed investigations that are beyond the scope of this study. 

### 2.3. Emission Spectra 

**Figure 2:**
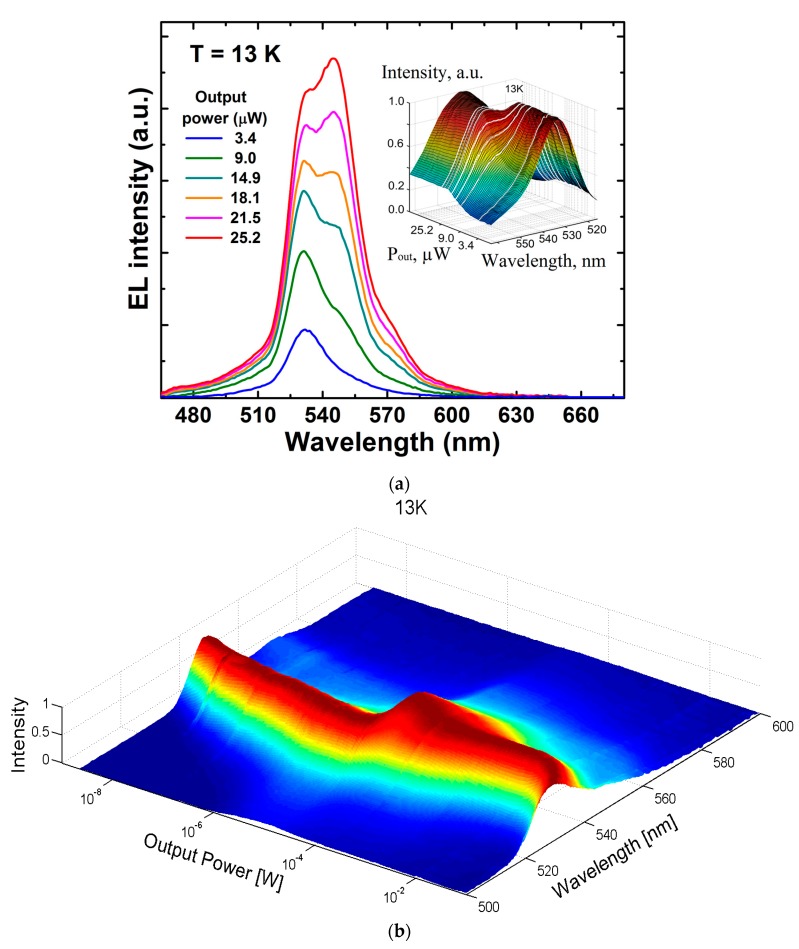

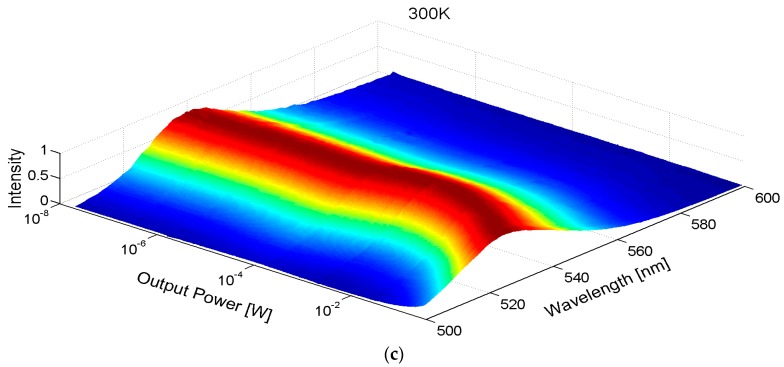
Emission spectra of the green LED at 13 K at various output power (**a**) where the insert shows these normalized spectra in 3D vision with additional one at 12 μW; and evolution of the normalized emission spectra vs. output power at 13 K (**b**) and 300 K (**c**).

The EL spectra of the green LED were monitored over a wide range of currents and temperatures. We found the spectra to consist of two main partly merged peaks (see Figure 2a). 

One of them, centered at about 535 nm and referred to hereafter as shorter-wavelength (sw) emission, was dominant at low output power. Another one centered at ~545 nm and referred to hereafter as longer-wavelength (lw) emission, became stronger at higher output power. The peaks competed with each other in a narrow range of power variation. This two-peak structure of the emission spectra was clearly seen at temperatures below 200 K and became blurred toward 300 K. A shoulder observed at ~565–570 nm (Figure 2a) was attributed to LO phonon replica, as it was red-shifted with respect to the lw-peak by the optical phonon energy of ~80–90 meV. A similar but much more pronounced LO phonon replica was observed previously in the emission spectra of InGaN/GaN quantum wells [5,17]. The shoulder was observed at temperatures lower than 100 K; at higher temperatures it could be resolved because of thermal broadening of the phonon replica.

In order to understand better the behavior of the EL spectra, we examined their normalized intensity vs. output power. For this purpose, 3D contour plots were generated using the Matlab programming framework. We processed and stored the measured EL intensity I as a function of wavelength λ and corresponding emission power *P* for each temperature. Spectra obtained in such a way were then normalized to a maximum intensity at every value of *P*. Then we created continuous normalized functions I(*P*, λ) using linear interpolation of the scattered data. The functions I(*P*, λ) obtained for cryogenic (13 K) and room (300 K) temperatures are plotted in Figure 2b,c using a log-spaced variable resolution along the P axis. 

Figure 2b demonstrates that transition from sw- to lw-emission peaks occurs in a very narrow range of optical power (transition region) from ~6 × 10^−6^ to ~5 × 10^−5^ W. Beyond this range both peaks exhibit blue shifts with growing *P* and overlap partly in the transition region. Similar evolution of the normalized emission spectra is observed at the temperatures from 13 K to 200 K. At temperatures over 200 K, however, the sw- and lw-peaks are almost indistinguishable in the normalized spectra, Figure 2c. Nevertheless, the transition region is represented via bowing of the EL peak towards longer wavelengths at *P* ~ 10^−3^–10^−2^ W. 

Summarizing the emission spectra behavior, one can see that the transition from the sw- to the lw-emission peak occurs in a narrow range of output power and has a “switching” character. Outside the narrow transition region, single sw- or lw-emission peaks dominate at low and high currents, respectively. 

### 2.4. Emission Efficiency 

**Figure 3:**
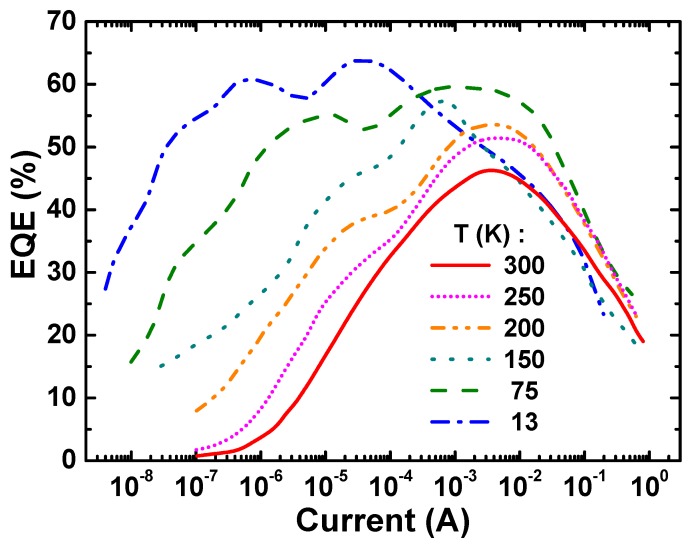
External quantum efficiency (EQE) of green LEDs versus driving current measured at various temperatures.

**Figure 4:**
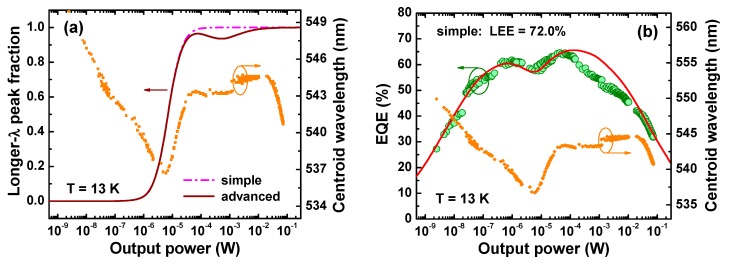

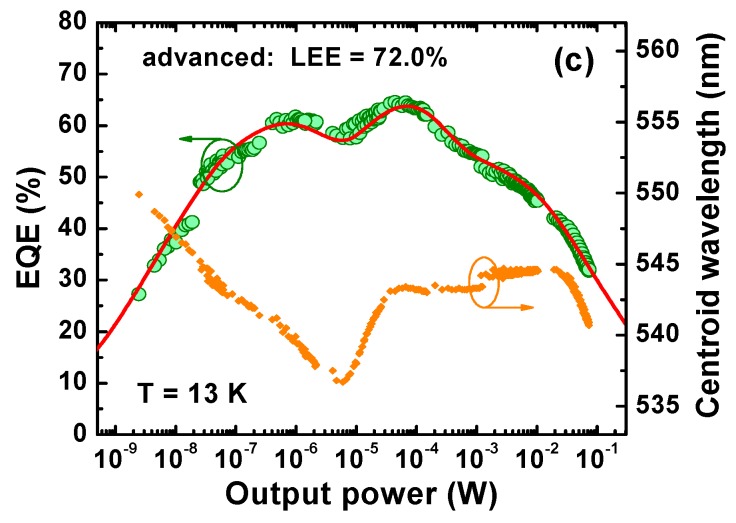
Simple and advanced transition functions used in the simulations (**a**), EQE of the green LED at *T* = 13 K predicted by modified ABC-model (solid lines) obtained with simple (**b**) and advanced (**c**) transition functions, and emission wavelength as a function of output optical power. Symbols are experimental data points.

**Figure 5:**
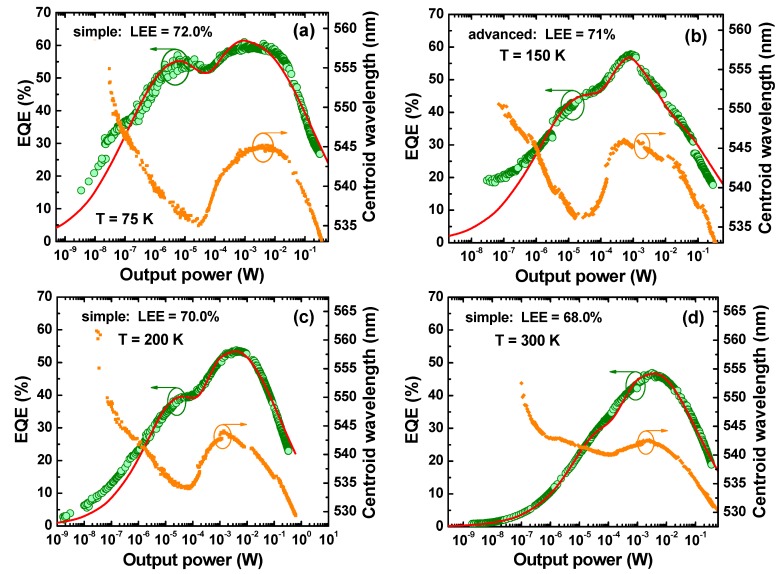
EQE of the green LED and emission wavelength as a function of output power measured at various temperatures (symbols) and predicted by modified ABC-model (solid lines). (**a**) Simple: LEE = 72.0%, T = 75 K; (**b**) advanced: LEE = 71%, T = 150 K; (**c**) simple: LEE = 70.0%, T = 200 K; (**d**) simple: LEE = 68.0%, T = 300 K.

The output power (*P*) and centroid wavelength measured at various temperatures versus operating current were converted to EQE and are presented in Figure 3. The same EQE data plotted vs. *P* are presented separately for every single temperature in Figure 4 and Figure 5. Looking at the low temperature EQE curves for 13 K and 75 K, one can see a distinct two-peak shape with local maxima located at notable different values of the current/output power. At higher temperatures above 75 K, the low-current peaks gradually transform to the shoulders and merge with high-current peaks. Unlike these two main peaks, the third one becomes apparent at low temperatures and low current only, ~0.1 µA. This behavior differs from one observed previously with blue LEDs which was an evenly symmetrical dome-like function EQE (*P*) on a log-scale. 

It is also important to note that the local minimum of EQE(*P*) is placed between two local maxima at every temperature and correlated well with transient region between two spectral peaks (see Figure 2) as well as with the red shift of centroid wavelength (Figure 4 and Figure 5). This observation allows us to attribute the low-current EQE peak to the sw-emission and high-current one to the lw-emission, respectively.

Since the measured two-peak EQE dependencies shown in Figure 4 and Figure 5 could not be approximated with a simple ABC-model, a new approach was developed for evaluation of IQE and LEE of the green LED. 

## 3. Modeling 

### 3.1. Model 

In our previous work [5] the IQE and LEE of a commercial blue LED were independently evaluated from measured EQE vs. forward current (I). The curve EQE(I) was fitted there with the ABC model using an original method based on the analytic expression for EQE as a function of the normalized optical power. The experimental multi-peak structure of the EQE dependence on current/output power shown in Figure 4b,c does not allow fitting by a simple ABC model, which predicts the efficiency to peak at a certain single value of the output power [18]. However, the model can be modified, assuming the properties of InGaN QWs in the LED active region (AR) to be non-uniform. The non-uniformity may be attributed to either InGaN composition or the QW width. From the efficiency point of view, various types of non-uniformity are possible. One is the vertical non-uniformity corresponding, for instance, to different InGaN compositions in top and bottom QWs. In this case, the overall IQE of the MQW active region *η_i_* can be regarded in the same manner as used for multi-color LEDs in [19] where

(1) $$\eta_{i}={\left(\sum_{k=1}^{N}f_{k}/\eta_{i}^{\left(k\right)}\right)}^{-1},$$

*f_k_* is the fraction of photons emitted by the *k*-th QW in the total LED emission spectrum and $\eta_{i}^{\left(k\right)}$ is IQE of the *k*-th well; summation in Equation (1) is performed over all *N* QWs. 

Another type of non-uniformity is the lateral/in-plane inhomogeneity. Here, different in-plane areas of the AR are assumed to emit light at different wavelengths and to have different IQEs, respectively. Assuming current density to be laterally uniform, one can come again to Equation (1) connecting *η_i_* with partial efficiencies $\eta_{i}^{\left(k\right)}$ and fractions of emitted photons *f_k_* associated with the above in-plane areas. For brevity, the QWs in the case of vertical non-uniformity and in-plane areas in the case of lateral non-uniformity will be referred to hereafter as different sub-regions (SRs), forming altogether the whole AR of the LED structure. 

Examination of the LED emission spectra (see Section 2.2) has revealed the existence of at least two SRs that emit at different wavelengths. As the LED operating current is increased, the sw-emission is nearly abruptly switched to the lw-emission (see Figure 2b,c). On the other hand, two efficiency peaks can be clearly seen in practically all the EQE plots vs. output optical power P shown in Figure 4b,c and Figure 5. It was already mentioned above that it is reasonable to attribute the two-peak character of EQE(*P*) curves to the existence of two SRs emitting at different wavelengths. 

As it has been suggested earlier for dual-wavelength LEDs [19], that the IQE of every particular SR, $\eta_{i}^{\left(k\right)}$, can be approximated by the analytical expression

(2) $$\eta_{i}^{\left(k\right)}=\frac{Q_{k}}{Q_{k}+{\left(P_{k}/P_{m}^{\left(k\right)}\right)}^{1/2}+{\left(P_{k}/P_{m}^{\left(k\right)}\right)}^{-1/2}};\;P_{k}=f_{k}P,$$

where *Q_k_* is the quality factor [18] of the *k*-th SR, $P_{m}^{\left(k\right)}$ is the optical power corresponding to the IQE maximum of the *k*-th SR, and *P_k_* is the optical power produced by the *k*-th SR at a chosen operating current. Within the ABC-model, *Q_k_* and $P_{m}^{\left(k\right)}$ are proportional to certain combinations of the recombination constants: *A* corresponding to Shockley-Read-Hall non-radiative recombination, *B* related to radiative recombination, and *С* associated with Auger recombination. So, if all *f_k_* are known, the total IQE of the LED structure *η_i_* can be calculated using Equations (1) and (2) and then parameters *Q_k_* and $P_{m}^{\left(k\right)}$ can be fitted to experimental data. 

Considering just two different SRs to coexist in the whole active region of green LEDs, one can regard the only fraction *f_lw_* corresponding to photons produced by SR with lw-emission; another fraction attributed to sw-emission is: $f_{sw}=1-f_{lw}$. As one can see from Figure 2b, switching between two spectral peaks occurs in a rather narrow range of the output power. In most of cases the switching could be well approximated by a simple “transition function”

(3) $$f_{lw}={\left[1+{\left(P_{t}/P\right)}^{\gamma }\right]}^{-1},$$

where *P_t_* is the critical output power corresponding to the transition between the efficiency peaks (transition power) and the parameter *γ* is related to the width of the transition region identified on the log-scale of the output power. We have found *γ* = 2 to fit well the transition from shorter to longer emission wavelength at temperatures from 13 K to 250 K. At 300 K, the value *γ* ~ 1.5–1.7 seems to be more suitable. Nevertheless, we used the parameter *γ* = 2 for 300 K as well in order to unify approximations of the spectral transition at various temperatures. 

The approximation based on Equation (3) will be called hereafter as “simple”. In some cases, non-monotonous transition functions should be applied to provide more accurate fitting of the characterization data. The latter type of the transition function will be referred to as “advanced”. In this study, we used the advanced transition function in the form

(4) $$f_{lw}=f_{1}\cdot {\left[1+{\left(P_{t}/P\right)}^{\gamma }\right]}^{-1}+\frac{f_{2}}{1+(P/P_{a})+(P_{a}/P)},$$

In the particular case presented in Figure 4c, the transition function (4) used the same parameters *P_t_* and *γ* as the simple transition function and additional parameters: *f*_1_ = 0.999998, *f*_2_ = 0.2, and *P_a_* = 0.5 mW. Parameters of the advanced transition function used for calculations shown in Figure 5b differ from the above ones by only *P_a_* = 7.0 mW. 

### 3.2. Model Application 

Assuming the light extraction efficiency (LEE) of LED to be the same for the close spectral peaks, we have fitted EQE(*P*) curves with Equations (1)–(3). For this purpose, parameters *Q_k_*, $P_{m}^{\left(k\right)}$ (*k* = sw, lw), and *P_t_* were first fitted to provide the best correlation with the multiple-peak dependence of LED efficiency on the output power. Then IQE was calculated using Equation (1). Finally, LEE was estimated as a ratio of experimental EQE and calculated IQE. 

The dash-dotted line in Figure 4a shows the simple switching function used for fitting of the EQE(*P*) measured at *T* = 13 K. The transition power *P_t_*, being the only adjustable parameter of the transition function, was determined from the observed “switching” in the LED emission spectra from sw- to lw-emission shown in Figure 2b. The use of the simple switching function with different *Q_k_* and $P_{m}^{\left(k\right)}$ corresponding to sw (*k* = sw) and lw (*k* = lw) peaks provided the theoretical curve shown by solid line in Figure 4b at LEE = 72%. The curve fits well the general shape of the EQE(*P*) dependence but fails in reproducing some of its details at output power between 10^−4^ W and 10^−2^ W. On the other hand, the spectral evolution of the emission spectra shown in Figure 2b is more complex than it is predicted by the simple transition function. Therefore, an advanced transition function (solid line in Figure 4a) has been applied. This enabled much better fitting of the experimental results, as one can see from Figure 4c. 

Figure 5 presents results of fitting EQE(*P*) measured at other temperatures. Except for that of 150 K, simple switching functions (4) were sufficient to get a reasonable fitting of the experimental points. As one can see from the figure, noticeable discrepancy between the fitting and the data is still observed at low power and *T* = 75–200 K. This may be the evidence for additional AR non-uniformity, manifesting itself just at extremely low currents. However, we will ignore this possibility in the further discussion, as no evidence for existence of one more distinct spectral peak was found. 

## 4. Discussion 

This section discusses general trends in variation of important LED characteristics and parameters with temperature. In order to interpret the trends, we will compare the results obtained for true-green LEDs with those reported earlier for blue LEDs [5]. 

### 4.1. Light Extraction Efficiency 

**Figure 6:**
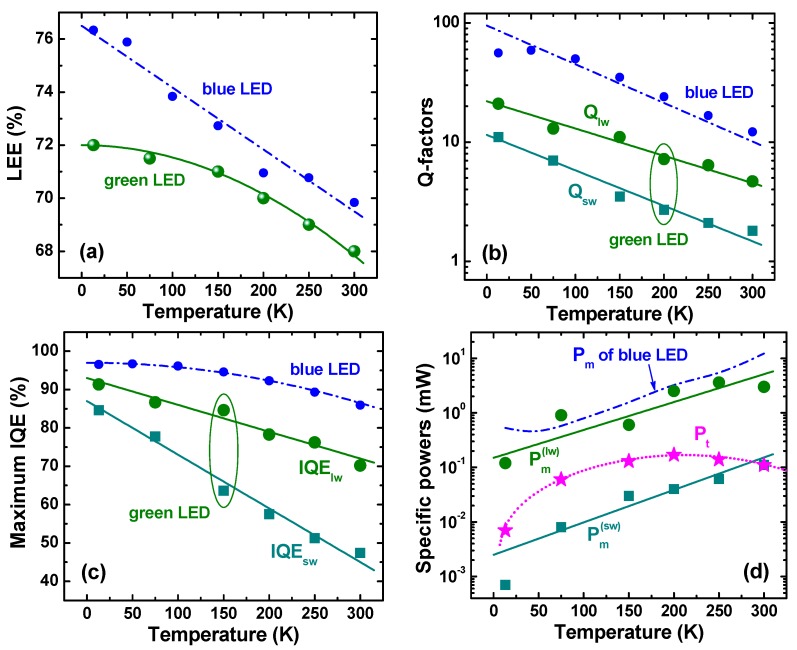
Light extraction efficiency (**a**); quality factors corresponding to lower-wavelength (*Q*_lw_) and shorter-wavelength (*Q*_sw_) spectral peaks (**b**); Maximum internal quantum efficiency (**c**); and specific optical power (see text for more detail) as a function of temperature given by big symbols (**d**). Dash-dotted lines and small circles show for comparison similar data reported earlier for blue LEDs [5]. Solid and dotted lines in the plots are drawn for eye.

Figure 6a compares the temperature-dependent efficiencies of light extraction from the LED dice to air obtained for green and blue LEDs. As one can see from Figure 6, the LEE of the green LED is lower than that of the blue one at all the temperatures. This finding disagrees with the increase in RT LEE from the blue to green spectral range observed on SQW-based LEDs [6]. The LEE increasing with wavelength was attributed in [6] to temperature-dependent optical properties of the silver-based *p*-electrode providing dominant optical losses of the emitted light via its incomplete reflection from the electrode. On the other hand, contribution of internal optical losses in the LED die was also found to be considerable. Nevertheless, in LED structures of similar designs, the optical losses have been shown not to change the general trend of the LEE to rise with the emission wavelength [6]. 

The AR of the green LED differs from those studied in [6] in two aspects. First, it consists of five InGaN QWs, which makes band-to-band light absorption quite critical for achieving high LEE. Secondly, the AR non-uniformity of the green LEDs results in two different spectral peaks (Section 2). Regarding that, the sw-emission may be effectively absorbed by the SR producing the lw-emission, which may increase additionally the band-to-band optical losses. As the sw-emission is more pronounced at low temperatures (*T* < 100–120 K), this should lead to a greater difference between LEEs of blue and green LEDs in this temperature range, in accordance with the data shown in Figure 6b. At high temperatures (*T* ~ 250–300 K), the sw-emission is suppressed and the difference between LEEs of blue and green LEDs in this temperature range (less than ~2%) may be attributed to a difference in the band-to-band light absorption in the MQW active region. 

Figure 5d shows that the RT EQE dependence on the output power plotted in log scale has a nearly dome-like character with asymmetric low-power and high-power wings. In this case, the procedure of the LEE extraction from this dependence based on approximation of the high-power wing with the ABC model [6] becomes applicable, providing the LEE value of about 66%. The method used in our study, which implies two ARs to co-exist, gives the value of 68% which is quite close to the above cruder estimate. 

### 4.2. Quality Factors and Internal Quantum Efficiency 

It has been shown in Section 3.2 that dependence of the EQE of the green LED on output optical power can be interpreted in terms of two co-existing ARs having different quality factors, *Q*_sw_ and *Q*_lw_, the temperature dependences of which is given in Figure 6b. One can see from the figure that *Q*-factors of the green LED are much lower than those reported in [5] for the blue one. This is manifestation of the so-called “green gap” problem, i.e., remarkable decline of the LED efficiency towards longer emission wavelength. Two commonly discussed origins of the “green gap” are: (i) quantum-confined Stark effect leading to spatial separation of electron and hole wave functions inside the InGaN QWs (see, for example, [2,3] and references therein) and (ii) degradation of materials quality in InGaN alloys with high indium content [20,21]. Recently, implication of composition fluctuations in InGaN to the LED efficiency reduction in the “green gap” has been also demonstrated [22,23,24]. 

Figure 6b shows that the *Q*-factor corresponding to sw-emission from the green LED is a few times lower than that corresponding to the lw-emission. Since the *Q*-factors determine the maximum IQE values through the relationship $IQE_{k}=Q_{k}/(Q_{k}+2)$ (*k* = sw, lw), the lower *Q*_sw_ results immediately in a lower IQE value of the sw-emission. The temperature dependencies of maximum IQEs for sw- and lw-emission of the green LED are plotted in Figure 6c. One can see from the figure that the efficiency of the sw-emission declines with temperature much faster than that of the lw-emission, resulting in suppression of the sw-peak in the emission spectrum at RT. In turn, the IQE corresponding to the lw-emission of the green LED declines faster than the efficiency of the blue LED. This observation is in line with the conclusion made in [23] that the contribution of Auger recombination to the temperature-dependent IQE reduction is much stronger in green LEDs than in blue ones.

Figure 6c shows also that absolute maximum of IQE is controlled by the lw-emission irrespective of temperature. The maximum IQE tends to the value of ~92% at zero temperature, i.e., the non-radiative recombination does not vanish completely at cryogenic temperatures. This makes doubtful estimations of the RT IQE based on comparison of the LED emission intensities measured at cryogenic and room temperatures.

### 4.3. Specific Powers and Recombination Volumes 

Output power corresponding to the maximum IQEs of the sw- ($P_{m}^{\left(sw\right)}$) and lw-emission ($P_{m}^{\left(lw\right)}$) of green LEDs are plotted in Figure 6d versus temperature along with the transition power *P_t_*. The only temperature-dependent output power *P_m_* of blue LEDs from [5] is also shown in the figure by the dash-dotted line just for comparison. One can see that $P_{m}^{\left(lw\right)}$ and *P_m_* of blue LEDs are rather close to each other in the whole temperature range of study. In contrast, $P_{m}^{\left(sw\right)}$ is about two orders of magnitude lower than $P_{m}^{\left(lw\right)}$ and *P_m_*. The reason for this is discussed below in more detail. 

The transition power *P_t_* is situated between $P_{m}^{\left(sw\right)}$ and $P_{m}^{\left(lw\right)}$ at *T* < 200 K and approaches $P_{m}^{\left(sw\right)}$ at RT. The latter corresponds to merging of the sw- and lw-emission peaks at *T* = 300 K, as can be seen from Figure 2c. 

**Figure 7:**
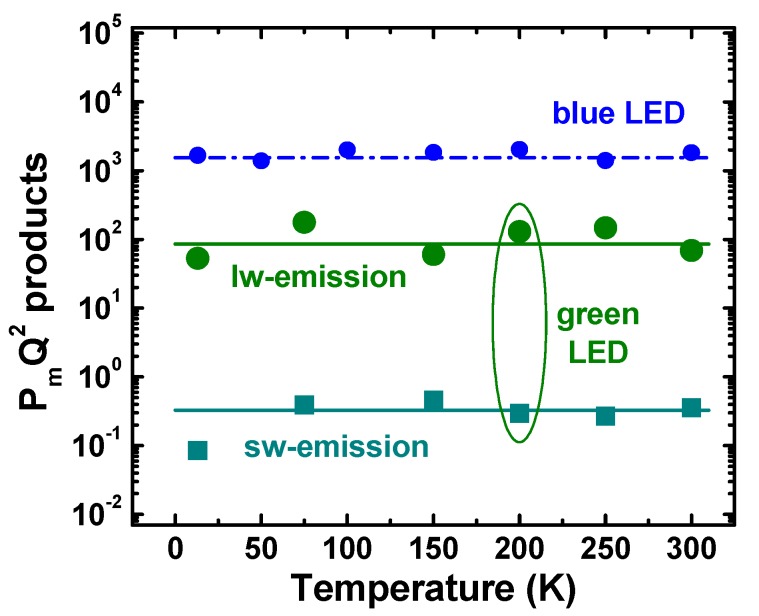
*P_m_Q*^2^ product as a function of temperature plotted for sw- and lw-emission of green LEDs. Big symbols are experimental points, solid lines are drawn for eyes. Data for blue LEDs reported in [5] are also shown for comparison by small circles and dash-dotted line.

In order to get more information from the data obtained, we have plotted in Figure 7 the $P_{m}Q^{2}$ products corresponding to sw- and lw-emission peaks vs. temperature. Since $P_{m}Q^{2}\propto E_{ph}V_{r}B^{3}/C^{2}$ where *E_ph_* is the energy of emitted photon, *V_r_* is the recombination volume, and *B* and *C* are the radiative and Auger recombination coefficients, respectively (see, for example, [18]), the $P_{m}Q^{2}$ product does not include the Shockley–Read–Hall coefficient *A* responsible for carrier recombination at point and extended defects. As one can see from Figure 7, the $P_{m}Q^{2}$ products of both sw- and lw-emission peaks are found to be nearly independent of temperature, similar to the case of blue LEDs [5]. This is evidence for the fact that both radiative and Auger recombination coefficients in the green LED, like in the blue one, have qualitatively similar temperature dependence: either ascending or descending. This result is in qualitative agreement with the recently observed anomalous (ascending) temperature variation of the radiative recombination coefficient [23], which was attributed to strong hole localization by composition fluctuations in InGaN alloys. Regarding that, the Auger recombination coefficients were found in [23] to increase with temperature in both blue and green LEDs. 

Using the values of the *B*- and *C*-coefficients at 450 nm and 540 nm reported in [25] for RT, we have estimated the ratio of the recombination volumes of blue LED ($V_{r}^{\left(b\right)}$) and that corresponding to lw-emission of green LED ($V_{r}^{\left(lw\right)}$). The obtained ratio $V_{r}^{\left(b\right)}/V_{r}^{\left(lw\right)}\approx 0.85$ indicates that the recombination volumes are comparable with each other. As the active region of the blue LED was specially optimized to provide a uniform carrier injection in all five QWs in the active region, the latter fact enables attributing the lw-emission of the green LED to operation of its active region as a whole. 

A similar estimation provides $V_{r}^{\left(b\right)}/V_{r}^{\left(sw\right)}\approx 220$, which demonstrates the recombination volume of the SR responsible for sw-emission to be about two orders of magnitude smaller than that of the SR producing lw-emission. Such a big difference cannot be explained by dominant carrier injection in one of the five QWs. Therefore, a natural interpretation of the result implies that the sw-emission comes from the local lateral areas distributed within the QWs. 

### 4.4. Possible Origins of Active Region Non-Uniformity 

Any interpretation of the above results should account for and/or explain four main points: (i) the difference between the wavelengths of sw- and lw-emission; (ii) the sequence of sw- and lw-peaks’ appearance in the emission spectrum with the operating current; (iii) the difference in the recombination volumes associated with sw- and lw-emission; and (iv) the difference in the *Q*-factors or IQEs of the sw- and lw-emission. We will consider below a number of scenarios for the active region non-uniformity, addressing these points. 

The difference in the emission wavelength can be attributed to variation of either composition or width of InGaN QWs in the LED active region. Simulations of InGaN SQW LED structures operating at the current density of 20 A/cm^2^ carried out with the commercial SiLENSe 5.10 package [26] have shown that the observed difference between the sw- and lw-emission wavelengths may be associated with either ~2% variation of the indium content in the InGaN alloy or ~0.4 nm (i.e., ~1.5 monolayer) variation of the QW width. Both versions seem to be realistic for green LEDs examined here. 

A possible mechanism producing compositional non-uniformity of the active region is partial stress relaxation in the LED structure. Indeed, the critical thickness of the (0001) InGaN/GaN layer is comparable with the QW widths in the green spectral range [27]. If the total width of all QWs in the LED active region exceeds the critical thickness, stress relaxation becomes possible, resulting, in particular, in an increase of indium content just by a few percent in the top partially relaxed QWs [28]. The appearance of the sw-emission at low currents and of lw-emission at high currents may be then explained, assuming dominant hole injection into the bottom (unrelaxed) QWs at low currents. This assumption does not agree, however, with the data of experiments carried out on LEDs with color-coded blue/cyan QWs in the active region [29]. In addition, the above scenario cannot explain a lower IQE of the sw-emission, as compared to lw-emission, and the substantial difference in their recombination volumes. 

A similar conclusion can be made about another mechanism, which implies the lw-emission to come from the In-rich clusters more or less uniformly distributed over all the QWs. Here, lw-emission is expected to appear first at low currents and its recombination volume should be smaller than that of the sw-emission, contrary to observations. Two-peak emission spectra may also be explained by contribution of excited states of electrons and holes in InGaN QWs. However, the expected sequence of lw- and sw-peak appearance in the spectra under increase of operating current (lw-peak at low currents and sw-peak at high currents) contradicts our observations as well. Therefore, other mechanisms are necessary in order to interpret the characterization data of our green LEDs. 

Here, we suggest the following qualitative model for the active region non-uniformity. First of all, we assume rarely distributed regions with lower indium content responsible for sw-emission to be embedded into the matrix of InGaN with higher indium content, which is responsible for lw-emission. These low-indium regions have a lower height of the potential barriers formed at the InGaN/GaN interfaces for both electrons and holes. This is due to lower band offsets in both conduction and valence bands and lower polarization charges induced at the interfaces. At low LED operating currents, the carrier injection into the InGaN QWs is limited by thermionic emission over the barriers. Therefore, pinching of the current is expected to occur in such a way, as to produce dominant pumping of the low-indium regions and, eventually, the sw-emission of photons. At higher currents, pumping of the high-indium matrix starts to occur, resulting in the lw-emission, which becomes quickly dominant, partly due to a much larger recombination volume. 

Such a scenario is consistent with most of observations discussed above but it does not yet explain the difference in the IQEs (*Q*-factors) of the sw- and lw-emission regions. The explanation can be given, assuming the low-indium regions to be formed around extended defects like threading dislocations and V-pits. Indeed, less effective indium incorporation is expected next to the dislocation cores because of excess elastic energy related to dislocation-mediated strain. Formation of the InGaN QWs with larger bandgap on the side walls of V-pits has been directly demonstrated in [30]. Existence of dislocation cores serving as non-radiative recombination centers may explain a lower IQE of the sw-emission regions. 

In order to assess whether the small recombination volume $V_{r}^{\left(sw\right)}$ may be attributed to extended defects, we assume the sw-emission to originate from the carriers collected from the area of about $L_{d}^{2}=D_{a}\tau_{d}$ around each of the defects, where *L_d_* is the carrier diffusion length, *D_a_* is the ambipolar diffusion coefficient, and *τ_d_* is the differential carrier life time. Using experimental values *D_a_* ~ 0.25 cm^2^/s [31] and *τ_d_* ~ 20 ns [23], typical for green InGaN LEDs operating at low currents, we obtain $L_{d}^{2}$ ~ 5 × 10^−9^ cm^2^. In this case, the density of the extended defects necessary to provide the ratio $V_{r}^{\left(b\right)}/V_{r}^{\left(sw\right)}\sim 220$ is ~10^6^ cm^−2^, which may be tentatively associated with the density of V-pits. Indeed, the carrier injection into semi-polar QWs formed at the side walls of V-pits is not hindered by high potential barriers typically induced at the (0001)-interfaces of polar QWs. Therefore, the current flow through the V-pits may dominate under low-current conditions [32]. This conclusion is indirectly supported by the experimental correlation between the waving observed in the I–V curves, which is the evidence for carrier leakage through extended defects, and the values of the current corresponding to maxima of the sw-emission efficiency (closed circles in Figure 1). Of course, the V-pit density of ~10^6^ cm^−2^ is just the lower-limit estimate, which does not account for the complex mechanism of current pinching around this type of defect [32] and dispersion of their dimensions affecting the current flow as well. 

It is interesting that the two-peak character of the low-temperature efficiency dependence on current has been reported earlier for both blue and green LEDs [7]. Moreover, the efficiency maxima were shifted remarkably to lower currents in green LEDs compared to blue ones, in line with our observations. Those data may also be interpreted in terms of competition between the current flow through the V-pits and through the (0001)-interfaces of QWs, assuming the efficiency of the main QWs to be somewhat lower than that of the semi-polar QWs on the side walls of the pits. At RT, the low-current efficiency peaks quenched and the efficiency dependence on current gains a conventional dome-like shape [7]. 

Generally, the above mechanism considering V-pits as the origin of AR non-uniformity is expected to be especially pronounced in green LEDs, as higher indium content in the QWs is known to favor V-pit formation. 

## 5. Conclusions 

In this study, we have observed mutually correlated, non-ordinary evolution of the LED emission spectra and efficiency which, to our best knowledge, had not been reported before. The observation could be interpreted in terms of the active region non-uniformity, assuming co-existence of at least two sub-regions emitting at different wavelengths and having different radiative efficiencies. The use of the ABC model extended to the case of a non-uniform active region enabled estimating the recombination volumes corresponding to these sub-regions, which were found to differ by a factor of ~220. To explain such a big difference, as well as the sequence of appearance of the sw- and lw-emission in the integral spectra, one of the sub-regions was associated with extended defects, like V-pits, whereas another sub-region was attributed to the main part of the LED’s active region. As higher indium content in InGaN QWs favors V-pit formation during LED structure growth, the above observations should be regarded as those specific for green LEDs and much less typical for blue ones. 

Characterization of the green LED in a wide temperature range from 13 K to 300 K allowed evaluating temperature-dependent LEE and IQE. The estimated efficiency of light extraction, 68% at RT, was slightly lower than that reported earlier for blue LEDs but it tended to saturate at low temperatures at the value of 72%, which was tentatively related to re-absorption of the sw-emission by the lw-emission sub-region. The maximum IQE of the green LED was found to decrease from ~91% at 13 K to ~70% at 300 K, and was controlled by the lw-emission from the main part of the active region. Remarkable deviation of the IQE maximum from 100% at cryogenic temperatures indicates that non-radiative recombination does not vanish in the green LED, in contrast to blue one where it was ~97% [5]. This makes doubtful the method of IQE determination based on comparison of EL intensity at cryogenic and room temperatures. 

Defect-mediated sw-emission approaches its maximum efficiency at very low currents, producing an additional peak/shoulder in the EQE dependence on current. In previous studies, it looks like the IQE of the defect-mediated emission exceeded that of the main active region. This resulted in a strong shift of the total IQE/EQE maximum of green LEDs to lower currents, as compared to the case of blue LEDs. In our study, the lw-emission from the main active region dominated at all temperatures. Therefore, the IQE/EQE maximum of the green LED is approached at nearly the same currents as in the case of blue LEDs examined in [5]. 

Processing of the characterization data has shown that the $B^{3}/C^{2}$ ratio, where *B* is the radiative and *C* is the Auger recombination coefficients, is nearly independent of temperature in both cases of sw- and lw-emission of green and blue LEDs. Accounting for rather strong temperature dependence of single *B* and *C* coefficients reported in [23], this fact implies existence of a universal relationship between the recombination coefficients in InGaN QWs irrespective of their particular composition. The physics behind this observation remains an open question to be addressed by future studies. 

## Abbreviations

The following abbreviations are used in this manuscript:

AR	Active region
EQE	External quantum efficiency
EL	Electroluminescence
IQE	Internal quantum efficiency
LED	Light-emitting diode
LEE	Light extraction efficiency
LO	Longitudinal optical
MQW	Multiple quantum well
ND	Neutral density
QW	Quantum well
RT	Room temperature
SQW	Single quantum well
WPE	Wall plug efficiency
